# CXCL8 Promotes Endothelial-to-Mesenchymal Transition of Endothelial Cells and Protects Cells from Erastin-Induced Ferroptosis via CXCR2-Mediated Activation of the NF-κB Signaling Pathway

**DOI:** 10.3390/ph16091210

**Published:** 2023-08-25

**Authors:** Hai-zhou Ji, Li Chen, Mi Ren, Sang Li, Tong-yu Liu, Hong-ju Chen, Hui-hui Yu, Yang Sun

**Affiliations:** 1 Department of Gynecology, Clinical Oncology School of Fujian Medical University, Fujian Cancer Hospital, Fuzhou 350014, China; doctor_jhz@fjmu.edu.cn (H.-z.J.); lichen@fjzlhospital.com (L.C.); 13559177333@163.com (S.L.); liu_tongyu@fjmu.edu.cn (T.-y.L.); f3000chen@sina.com (H.-j.C.); yuhui101418@163.com (H.-h.Y.); 2 Department of Oncological Nursing, Clinical Oncology School of Fujian Medical University, Fujian Cancer Hospital, Fuzhou 350014, China; 13665069981@163.com

**Keywords:** CXCL8, CXCR2, NF-κB, ferroptosis, endothelial cells

## Abstract

CXCL8-CXCR1/CXCR2 signaling pathways might form complex crosstalk among different cell types within the ovarian tumor microenvironment, thereby modulating the behaviors of different cells. This study aimed to investigate the expression pattern of CXCL8 in the ovarian tumor microenvironment and its impact on both endothelial-to-mesenchymal transition (EndMT) and ferroptosis of endothelial cells. The human monocytic cell line THP-1 and the human umbilical vein endothelial cell line PUMC-HUVEC-T1 were used to conduct in vitro studies. Erastin was used to induce ferroptosis. Results showed that tumor-associated macrophages are the major source of CXCL8 in the tumor microenvironment. CXCL8 treatment promoted the nucleus entrance of NF-κB p65 and p65 phosphorylation via CXCR2 in endothelial cells, suggesting activated NF-κB signaling. Via the NF-κB signaling pathway, CXCL8 enhanced TGF-β1-induced EndMT of PUMC-HUVEC-T1 cells and elevated their expression of *SLC7A11* and *GPX4*. These trends were drastically weakened in groups with CXCR2 knockdown or SB225002 treatment. TPCA-1 reversed CXCL8-induced upregulation of SLC7A11 and GPX4. CXCL8 protected endothelial cells from erastin-induced ferroptosis. However, these protective effects were largely canceled when *CXCR2* was knocked down. In summary, CXCL8 can activate the NF-κB signaling pathway in endothelial cells in a CXCR2-dependent manner. The CXCL8-CXCR2/NF-κB axis can enhance EndMT and activate *SLC7A11* and *GPX4* expression, protecting endothelial cells from ferroptosis.

## 1. Introduction

CXCL8 (interleukin-8, IL-8) is a pro-inflammatory chemokine that binds to and activates two G protein-coupled receptors, CXCR1 and CXCR2 [1]. It is known that different cell types in the ovarian cancer tumor microenvironment might secrete CXCL8, including tumor-associated macrophages, neutrophils, and endothelial cells [2,3]. In addition, CXCR1 and CXCR2 are also expressed in different cells, such as tumor cells, tumor-associated mesenchymal stromal cells (MSCs), and endothelial cells [4,5]. Therefore, CXCL8-CXCR1/CXCR2 signaling pathways through autocrine and paracrine mechanisms form complex crosstalk among the cells in the ovarian tumor microenvironment. For example, CXCL8 initiates the epithelial-mesenchymal transition (EMT) program and activates Wnt/beta-catenin signaling in ovarian cancer cells, promoting cancer cell invasion and metastasis [5]. CXCL8 can recruit tumor-associated neutrophils, which partly impair the cytotoxic effects of CD8+ T cells in a contact-dependent manner [3]. In addition, this signaling axis promotes angiogenesis, which supports tumor growth and metastasis in ovarian cancer [6,7]. Targeting CXCR1 and CXCR2 has shown promising results in preclinical studies as a potential therapeutic strategy for cancer treatment. Therefore, understanding CXCL8-CXCR1/CXCR2 signaling pathways in the ovarian tumor microenvironment could provide theoretical support for targeted therapies.

The effect of CXCL8 on promoting angiogenesis in ovarian tumor tissues has been well characterized in previous studies [8,9]. Endothelial cells are a crucial component in the ovarian tumor microenvironment [10]. They are essential for angiogenesis, which supports tumor growth and metastasis [11]. Prevention of neovascularization is a critical target for ovarian cancer therapy. Although the utilization of some angiogenetic inhibitors, such as bevacizumab and tyrosine kinase inhibitors (TKIs), has shown some therapeutic benefits, acquired drug resistance will eventually lead to therapeutic failure [12]. Besides, endothelial cells can undergo endothelial-to-mesenchymal transition (EndMT), an important source of cancer-associated fibroblasts (CAFs) in the tumor microenvironment [13]. NF-κB signaling pathway is activated during EndMT [14,15] and promotes the transition [16].

Recent studies have shown that ferroptosis can have anti-angiogenic effects by inducing endothelial cell death, thereby inhibiting angiogenesis [17,18]. Ferroptosis is a form of programmed necrosis that involves the accumulation of reactive oxygen species (ROS) within cells in an iron-dependent manner [19]. This process can be induced by the small-molecule compound erastin, which inhibits the activity of the cystine-glutamate antiporter SLC7A11 (also known as system Xc−), ultimately causing a depletion of glutathione (GSH) [19]. Inhibiting CXCR2 might trigger ferroptosis in breast cancer cells [20], suggesting a potential role of the CXCL8-CXCR2 axis in ferroptosis. In this study, we further explored the expression profile of CXCL8 in the tumor microenvironment and the potential mechanisms of endothelial cell ferroptosis.

## 2. Results

### 2.1. Tumor-Associated Macrophages Might Be the Major Sources of CXCL8 in the Ovarian Tumor Microenvironment

**Figure 1:**
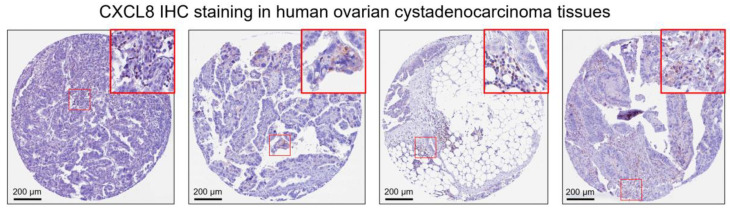
Representative images of IL-8 IHC staining in the human ovarian cystadenocarcinoma tissues. Images were retrieved from: https://www.proteinatlas.org/ENSG00000169429-CXCL8/pathology/ovarian+cancer#ihc accessed on 10 May 2023.

To characterize the expression profile of CXCL8 in the ovarian cancer tumor microenvironment, we checked immunohistochemistry (IHC) staining in the human protein atlas (HPA). Results showed that tumor cells usually have negative CXCL8 staining (Figure 1). However, a small proportion of immune cells showed positive CXCL8 expression (Figure 1, red frames).

**Figure 2:**
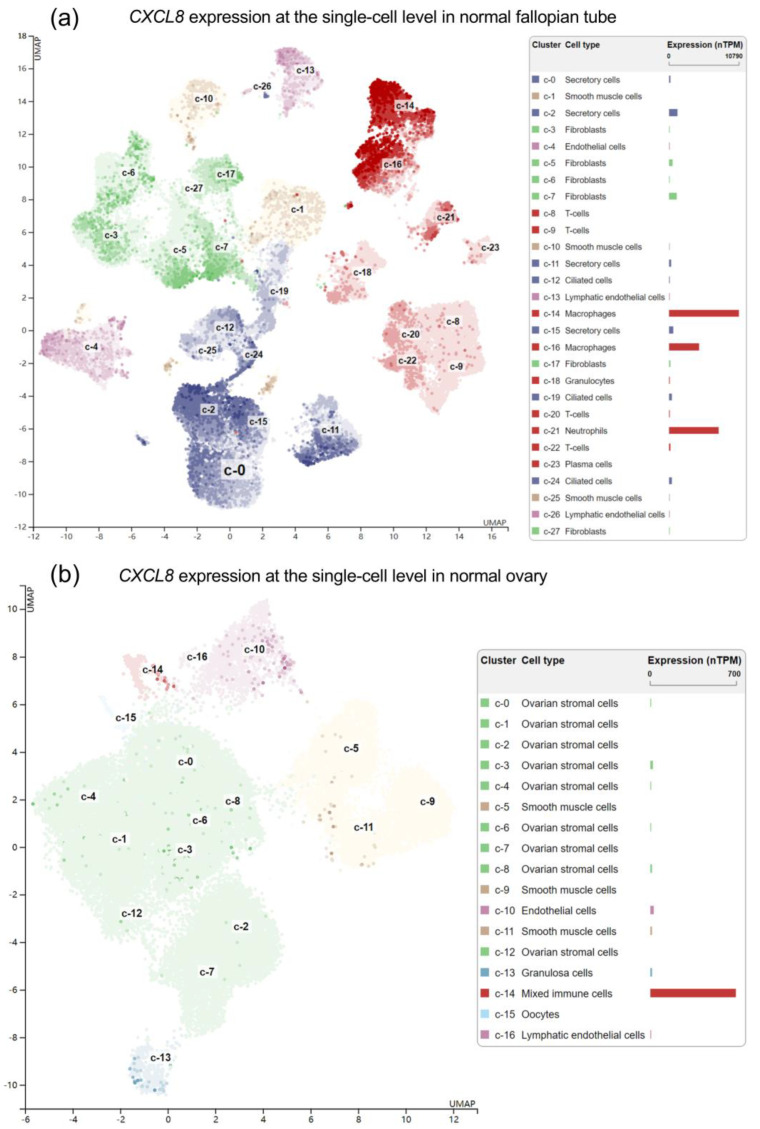
CXCL8 expression at the single-cell level in normal human fallopian tubes (**a**) and ovarian tissue. (**b**) Data were retrieved from: v22.proteinatlas.org, https://www.proteinatlas.org/ENSG00000169429-CXCL8/single+cell+type/fallopian+tube and https://www.proteinatlas.org/ENSG00000169429-CXCL8/single+cell+type/ovary accessed on 6 August 2023.

It is generally accepted that high-grade serous ovarian adenocarcinoma is derived from the fallopian tube [21,22]. To identify the specific immune cells with positive *CXCL8* expression, we analyzed single-RNA-seq data in normal human fallopian tubes and ovarian tissues in the HPA. Results indicated that macrophages are the dominant source of *CXCL8* (Figure 2a).

**Figure 3:**
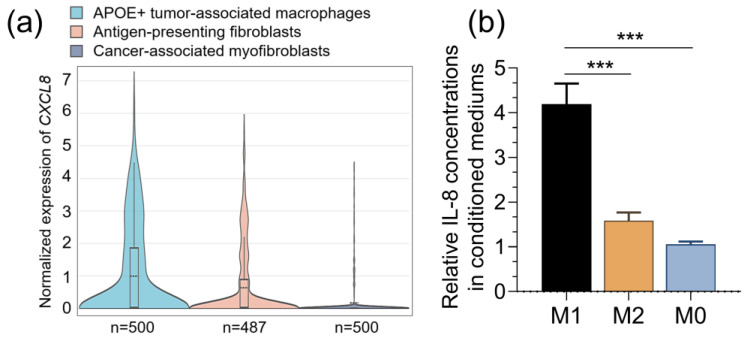
(**a**). CXCL8 expression at the single-cell level in normal human cancer-associated fibroblasts and macrophages. Data were retrieved from: https://gist-fgl.github.io/sc-caf-atlas/. (**b**). An ELISA was performed to determine the concentration of CXCL8 (IL-8) in the conditioned medium from M2, M1, and M0 macrophages. ***, *p* < 0.001.

To explore the specific macrophages with *CXCL8* expression, we reviewed another recent single-cell RNA-seq dataset focusing on pan-cancer immune cells and fibroblasts [23]. Results showed that tumor-associated macrophages and antigen-presenting fibroblasts have significantly higher *CXCL8* expression than cancer-associated myofibroblasts (Figure 3a). These findings imply that M2 macrophages might be the dominant source of *CXCL8* in the ovarian tumor microenvironment. To validate this finding, we developed M0, M1, and M2 macrophages from THP-1 cells. Then, an ELISA was performed to quantify the concentration of CXCL8 in the conditioned medium from these cells. Results showed that the conditioned medium from both M1 and M2 macrophages had a significantly higher CXCL8 concentration than that from M0 macrophages (Figure 3b).

### 2.2. CXCL8 Treatment Activates the NF-κB Signaling Pathways via CXCR2 in Endothelial Cells

**Figure 4:**
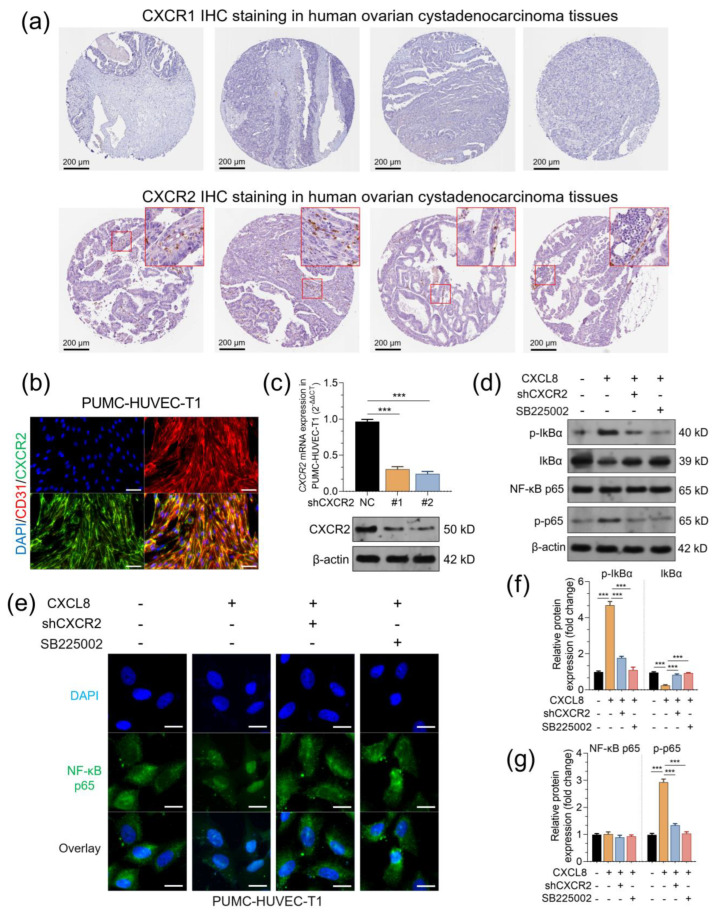
CXCL8 treatment activates the NF-κB signaling pathways via CXCR2 in endothelial cells. (**a**). Representative images of CXCR1 and CXCR2 IHC staining in human ovarian cystadenocarcinoma tissues. Images were retrieved from v22.proteinatlas.org, https://www.proteinatlas.org/ENSG00000163464-CXCR1/pathology/ovarian+cancer#ihc, and https://www.proteinatlas.org/ENSG00000180871-CXCR2/pathology/ovarian+cancer#img. (**b**). IF staining was performed to check CD31 (red) and CXCR2 (green) expression in PUMC-HUVEC-T1 cells. (**c**). qRT-PCR and western blotting were performed to check *CXCR2* expression in PUMC-HUVEC-T1 cells 48 h after lentivirus-mediated *CXCR2* knockdown. (**d**,**f**,**g**). Western blotting was performed to check the expression of IκBa, p-IκBa, NF-κB p65, and p-p65 expression in PUMC-HUVEC-T1 cells with CXCL8 treatment alone or in combination with SB225002 (30 nM, added 2 h before CXCL8 treatment) and in the cells with *CXCR2* knockdown and CXCL8 treatment (50 ng/mL for 12 h). Quantitation (*n* = 3, **f**,**g**) of protein expression was conducted based on integrated density measured by ImageJ. (**e**). Immunofluorescent staining was performed to visualize NF-κB p65 expression in PUMC-HUVEC-T1 cells as treated in panel (**d**). ***, *p* < 0.001.

It is known that CXCL8 regulates tumor behavior through its receptors and downstream signaling pathways. To characterize the expression profiles of CXCR1 and CXCR2 in the tumor microenvironment of ovarian cancer tumors, we checked IHC staining in the HPA. Results showed tumor cells usually have negative CXCR1 and CXCR2 staining (Figure 4a). CXCR1 is almost undetectable by IHC in all cell types in the sections (Figure 2a, top panel). However, a small proportion of immune cells (probably neutrophils, according to previous publications [3,24]) and endothelial cells presented positive CXCR2 expression (Figure 4a, bottom panel, red frames). We purchased commercial PUMC-HUVEC-T1 cells and confirmed CXCR2 expression on the cellular membrane by immunofluorescent staining (Figure 4b). Then, these cells were subjected to lentivirus-mediated *CXCR2* knockdown (Figure 4c). CXCL8 treatment (50 ng/mL for 12 h) significantly increased the expression of p-IκBα and p-NF-κB p65 (p-p65), suggesting an activated NF-κB pathway (Figure 4d,f,g). IF staining confirmed that CXCL8 treatment increased the nucleus accumulation of NF-κB p65 (Figure 4e). Knockdown of *CXCR2* or using a CXCR2 antagonist (SB225002, 30 nM, added 2 h before CXCL8 treatment) weakened or abrogated CXCL8-induced activation of the NF-κB signaling pathway (Figure 4d–g).

### 2.3. CXCL8 Promotes the Endothelial-Mesenchymal Transition via CXCR2

**Figure 5:**
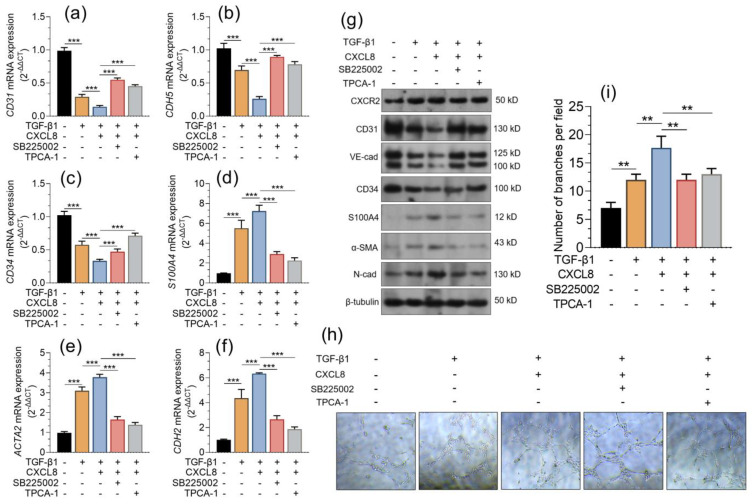
CXCL8 promotes EndMT in endothelial cells via CXCR2 (**a**–**g**). qRT-PCR (**a**–**f**) and western blotting (**g**) were performed to assess the expression of CXCR2, CD31, CDH5, CD34, S100A4, ACTA2, and CDH2 in n PUMC-HUVEC-T1 cells treated with TGF-β1 (5 ng/mL) for 24 h alone or in combination with CXCL8 (50 ng/mL). For the indicated groups, SB225002 (30 nM, a CXCR2 antagonist) or TPCA-1 (1 μM, a potent selective inhibitor of IKK-2) were applied 2 h before CXCL8 treatment. (**h**,**i)**. A tube formation assay was performed to assess the use of Matrigel (**h**), with treatments indicated in panel a. The number of tubule branches per view field was calculated using the angiogenesis analyzer of Image J software (v.1.52) (**i**). The data are expressed as the mean ± SD of three experiments. **, *p* < 0.01; ***, *p* < 0.001.

During endoMT induced by TGF-β1, although the expression of endothelial markers decreases [25], the cells gain stronger capability for tube formation [26,27]. Therefore, we explored whether CXCL8 contributed to TGF-β1 induced EndMT in PUMC-HUVEC-T1 cells. TGF-β1 increased CXCR2 expression. CXCL8 enhanced TGF-β1-induced downregulation of *CD31*, *CDH5,* and *CD34* and the upregulation of *S100A4*, *ACTA2,* and *CDH2* at the mRNA and protein levels (Figure 5a–g). However, these effects were largely canceled by SB225002 or TPCA-1 pretreatment (Figure 5a–g). The following tube formation assays confirmed that CXCL8 had an additive effect on TGF-β1-promoted tube formation (Figure 5h,i). Similarly, SB225002 or TPCA-1 pretreatment weakened CXCL8-induced tube formation of PUMC-HUVEC-T1 cells (Figure 5h,i).

### 2.4. CXCL8 Stimulates the Expression of SLC7A11 and GPX4 via the CXCR2-Mediated NF-κB Signaling Pathway

**Figure 6:**
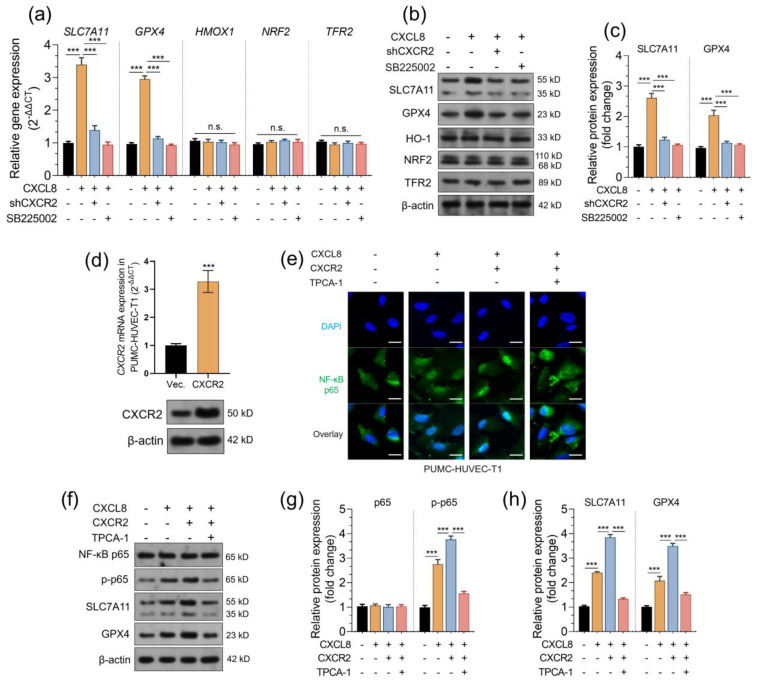
CXCL8 stimulates the expression of SLC7A11 and GPX4 via the CXCR2-mediated NF-κB signaling pathway. (**a**–**c**). qRT-PCR (**a**) and western blotting (**b**,**c**) were performed to check the expression of ferroptosis-related genes, including SLC7A11, GPX4, HMOX1, NRF2, and TFR2, in PUMC-HUVEC-T1 cells with CXCL8 treatment alone or in combination with SB225002 (30 nM, added 2 h before CXCL8 treatment) and in the cells with CXCR2 knockdown and CXCL8 treatment (50 ng/mL for 12 h). (**c**) The changes in SLC7A11 and GPX4 were quantified based on the integrated density measured by ImageJ (n = 3). (**d**). qRT-PCR and western blotting were performed to check CXCR2 expression in PUMC-HUVEC-T1 cells 48 h after lentivirus-mediated CXCR2 overexpression. (**e**). Immunofluorescent staining was performed to visualize NF-κB p65 distribution in PUMC-HUVEC-T1 cells with CXCL8 treatment alone (50 ng/mL for 12 h) or in CXCR2-overexpressed PUMC-HUVEC-T1 cells with CXCL8 treatment alone (50 ng/mL for 12 h) or in combination with TPCA-1 (1 μM, added 2 h before CXCL8 treatment). (**f**–**h**). Western blotting was performed to check the expression of NF-κB p65, p-p65, SLC7A11, and GPX4 in PUMC-HUVEC-T1 cells as treated in panel e. Quantitation (n = 3, **f**,**g**) of protein expression was conducted based on integrated density measured by ImageJ. n.s., not significant; ***, *p* < 0.001.

Since NF-κB signaling has known regulatory effects on ferroptosis-related genes, such as SLC7A11 and GPX4 [28,29], we decided to investigate whether CXCL8 regulates the expression of ferroptosis-related genes via the CXCR2-mediated NF-κB signaling pathway. CXCL8 treatment significantly elevated the transcription and translation of SLC7A11 and GPX4 but not HMOX1, NRF2, or TFR2 (Figure 6a,b). However, these trends were drastically weakened in groups with CXCR2 knockdown or SB225002 treatment (Figure 6a–c). To further validate that these alterations are mediated by the NF-κB signaling pathway, we overexpressed CXCR2 in PUMC-HUVEC-T1 cells (Figure 6d). Compared to the vector control, cells with CXCR2 overexpression have increased nucleus accumulation of p65 (Figure 6e) and higher expression of p-p65 (Figure 6f,g). However, when TPCA-1 (1 μM) was applied, CXCL8-induced p65 phosphorylation and nucleus translocation were inhibited (Figure 6e–g). In addition, TPCA-1 treatment also reversed CXCL8-induced upregulation of SLC7A11 and GPX4 (Figure 6f,h).

### 2.5. CXCL8 Protects Endothelial Cells from Erastin-Induced Ferroptosis

**Figure 7:**
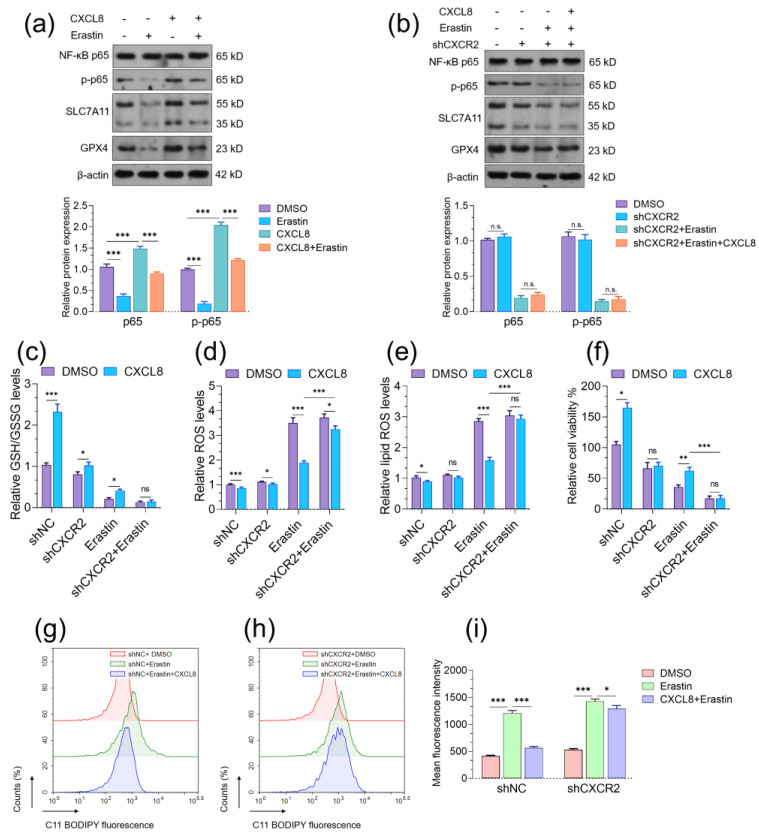
CXCL8 protects endothelial cells from erastin-induced ferroptosis. (**a**,**b**). Western blotting was performed to check the expression of NF-κB p65 and p-p65 in PUMC-HUVEC-T1 cells with CXCL8 treatment alone (50 ng/mL for 24 h), erastin treatment alone (10 μM for 24 h) or in combination (**a**), and the CXCR2 knockdown cells with erastin treatment alone (10 μM for 24 h) or in combination with CXCL8 treatment (50 ng/mL for 24 h) (**b**). Quantitation (n = 3, bottom panels) of protein expression was conducted based on integrated density measured by ImageJ. (**c**–**f)**. GSH/GSSG ratios (**c**), ROS levels (**d**), Lipid ROS (**e**), and relative cell viability % (**f**) were measured in PUMC-HUVEC-T1 cells with the indicated treatment alone or in combination. CXCL8 treatment: 50 ng/mL for 24 h; erastin treatment: 10 μM for 24 h. (**g**–**i**). Lipid ROS production was assessed by flow cytometry using C11-BODIPY. n.s., not significant; *, *p* < 0.05; **, *p* < 0.01; ***, *p* < 0.001.

Since CXCL8 stimulates the expression of SLC7A11 and GPX4, we further explored its potential protective effects on erastin-induced ferroptosis. Erastin treatment significantly suppressed NF-κB p65 phosphorylation, which was partially rescued by CXCL8 treatment (Figure 7a). However, in cells with CXCR2 knockdown, CXCL8-induced rescue was canceled (Figure 7b). CXCL8 treatment increased the basal GSG/GSSG ratio (Figure 7c), reduced ROS (Figure 7d), and lipid ROS levels (Figure 4e,g,i), and enhanced cell viability (Figure 7f). In addition, it alleviated erastin-induced GSG/GSSG drop (Figure 5c), reduced erastin-induced ROS (Figure 7d) and lipid ROS (Figure 7e,g,i), and protected cell viability upon erastin treatment (Figure 7f). These protective effects of CXCL8 were abrogated in cells with CXCR2 knockdown (Figure 7c–i).

## 3. Discussion

Tumor-associated macrophages with protumor and inflammatory characteristics (VEGFhigh/CXCL8+/IL1β+) are found in solid ovarian tumors [30,31]. The expression and production of CXCL8 by macrophages were enhanced via reactive oxygen species (ROS) and NF-κB activation [32]. In this study, we explored the expression profile of *CXCL8* in the tumor microenvironment using previous databases and confirmed that tumor-associated macrophages are the major source of CXCL8. In addition, we validated upregulated CXCL8 secretion from polarized macrophages. Typically, the pro-inflammatory M1 macrophages had significantly stronger CXCL8 expression and secretion than M0 and M1 macrophages. These trends were consistent with previous findings [33,34].

The overexpression of *CXCR2* in tumor cells can lead to ovarian cancer progression by enhancing NF-κB activation via EGFR-transactivated Akt signaling, thereby upregulating the expression of pro-inflammatory chemokines [35]. In addition, the CXCL8-CXCR2 axis can stimulate the activation of the NF-κB pathway in THP-1 monocytes [36], which modulates GSH generation [29,37]. SB332235, a CXCR2 antagonist, can ameliorate thioacetamide-induced activation of the TNF-α and NF-κB signaling pathways and alleviate thioacetamide-induced elevation of serum nitric oxide (NO) and malondialdehyde (MDA) levels and downregulation of GSH and superoxide dismutase (SOD) levels in both brain and liver tissues of rats [38]. These findings imply that the CXCL8-CXCR2 axis might be an important upstream axis modulating the NF-κB signaling pathway. Therefore, we checked whether this is a generalized mechanism in endothelial cells. Our data confirmed that CXCL8 stimulates NF-κB p65 phosphorylation and nucleus entrance via CXCR2 in endothelial cells.

Oxidative stress is a major hallmark of cancer due to imbalanced ROS production and antioxidant defenses within the tumor microenvironment [39]. Cancer progression is also an adaptive process for cancer cells to acquire a stronger antioxidant capacity to deal with various oxidative damages [39]. EndMT generates up to 40% of CAFs in the tumor microenvironment [13,40]. CAFs can become potent supporters of ovarian carcinogenesis and promote the initiation of tumor growth, invasion, and metastasis [41,42]. In this study, we confirmed that CXCL8 could promote TGF-β1-induced EndMT via CXCR2 and the NF-κB signaling pathway. Recent studies have also linked CAFs and ferroptosis resistance to cancer [43,44]. Exosomal miR-522 from CAFs can inhibit ferroptosis in cancer cells by reducing ALOX15 expression and blocking lipid-ROS accumulation [43]. In addition, Thrombospondin-4 secreted by CAFs confers ferroptosis resistance in GBM cell lines by transcriptionally upregulating the expression of the lncRNA DLEU1 [44].

It was observed that erastin treatment at a non-lethal level could induce a ferroptosis-like phenotype that promotes endothelial cell activation. Under this status, endothelial cells acquire enhanced proliferation, migration, and tube formation capabilities [45]. In addition, this ferroptosis-like phenotype promotes the formation of vascular endothelial cadherin junctional gaps and supports cancer cell adhesion to endothelial cells and transendothelial migration [45]. Therefore, although inducing ferroptosis might be a novel strategy in tumor treatment [19], acquired resistance to ferroptosis might generate more malignant phenotypes of the tumors.

An activated NF-κB signaling pathway can protect cancer cells from ferroptosis by transcriptionally activating the expression of multiple ferroptosis-related genes [28,29,37]. In this study, we found that CXCL8 could stimulate the expression of *SLC7A11* and *GPX4* via CXCR2-mediated activation of the NF-κB signaling pathway. Epithelial ovarian cancer patients with high co-expression of *SLC7A11* and *GPX4* are predicted to have unfavorable survival and platinum resistance [46]. SLC7A11 is a subunit of the cystine-glutamate antiporter that regulates cystine and glutamate exchange across the cellular membrane. This process is critical for maintaining intracellular levels of glutathione, a key antioxidant that counteracts oxidative stress [47]. GPX4 is an enzyme that utilizes GSH as a cofactor to reduce lipid hydroperoxides and phospholipid hydroperoxides to their corresponding alcohols [47]. SLC7A11 and GPX4 are particularly important for protecting cells from ferroptosis. Our functional assays confirmed that CXCL8 protects endothelial cells from erastin-induced ferroptosis by alleviating erastin-induced GSG/GSSG drop and suppressing erastin-induced ROS and lipid ROS. However, these protective effects were largely canceled when *CXCR2* was knocked down.

## 4. Materials and Methods

### 4.1. Bioinformatic Analysis

To evaluate gene expression at the individual cell level, two RNA-seq datasets were analyzed, one from the Human Protein Atlas (HPA) [48] and another recently published dataset (Luo 2022) that focuses on cancer-associated fibroblasts and macrophages [14]. Additionally, protein-level gene expression was assessed by examining immunohistochemistry data from the HPA [49].

### 4.2. Cell Culture and Treatment

The human umbilical vein endothelial cell line PUMC-HUVEC-T1 and human monocytic cell line THP-1 cells were purchased from Procell (Wuhan, China). PUMC-HUVEC-T1 cells were maintained in DMEM (PM150210, Procell) + 0.01mg/mL Insulin + 1%NEAA (PB180424, Procell) + 10%FBS (164210, Procell) + 1%P/S (PB180120, Procell). THP-1 cells were maintained in RPMI-1640 Medium (Gibco) supplemented with 10% FBS. DMEM and RPMI-1640 mediums have penicillin (100 U/mL, Gibco) and streptomycin (100 μg/mL, Gibco). Cells were cultured in a cell incubator at 37 °C with a 5% CO2 supply.

To generate M0 macrophages, THP-1 cells were exposed to 10 ng/mL of 12-O-tetradecanoylphorbol-13-acetate (TPA) for 24 h. Following this, the medium containing TPA was replaced with fresh RPMI-1640 medium supplemented with 10% FBS and 20 ng/mL of human recombinant M-CSF. The cells were then incubated for an additional 48 h to obtain M0 macrophages. For the induction of M1 macrophages, M0 macrophages were stimulated with 20 ng/mL of human recombinant IFN-γ and 100 ng/mL of lipopolysaccharide (LPS) for an additional 24 h. On the other hand, to induce M2 macrophages, M0 macrophages were stimulated with 20 ng/mL of human recombinant IL-4 and 20 ng/mL of human recombinant IL-13 for an additional 24 h.

To evaluate the influence of CXCL8 on the endothelial-to-mesenchymal transition of PUMC-HUVEC-T1 cells, the cells were serum-starved overnight and then treated with TGF-β1 (5 ng/mL) for 24 h alone or in combination with CXCL8 (50 ng/mL). For the indicated groups, SB225002 (30 nM, a CXCR2 antagonist) or TPCA-1 (1 μM, a potent selective inhibitor of IKK-2) were applied 2 h before CXCL8 treatment. TGF-β1, SB225002, TPCA-1, and erastin (a ferroptosis inducer) were purchased from Selleck (Houston, TX, USA). Recombinant human CXCL8 protein (Cat# 208-IL) was obtained from R&D Systems (Minneapolis, MN, USA).

### 4.3. Immunofluorescence Staining

PUMC-HUVEC-T1 cells were cultured on coverslips in 24-well plates. Once the cells reached approximately 50% confluence, immunofluorescence staining was conducted following a previously described method [5]. The coverslips were incubated overnight at 4 °C with mouse anti-CD31 (1:500, 66065-2-Ig, Proteintech, Wuhan, China) and rabbit anti-CXCR2 (1:50, 20634-1-AP, Proteintech) or anti-NF-κB p65 (1:500, #8242, Cell Signaling Technology, Danvers, MA, USA) diluted in blocking buffer. Following incubation, the coverslips were washed and incubated with secondary Alexa Fluor 594 conjugated anti-mouse IgG and Alexa Fluor 488 conjugated anti-rabbit IgG (1:1000, #8890, and #4412, Cell Signaling Technology) for 1 h at room temperature. After washing, the nuclei were counterstained using a mounting medium containing DAPI. Fluorescent images were captured using a wide-field microscope (IX81, Olympus, Tokyo, Japan).

### 4.4. Lentiviral Infections

A lentivirus for knocking down *CXCR2* was generated using the pLKO.1-puro plasmid. The following validated shRNA sequences were used: shCXCR#1, 5′-CCCTGGAAATCAACAAGTATT-3′; shCXCR2#2, 5′- GCTGGTGTTGTTGAAAGATAT-3′. The following scramble sequence was used as a negative control: shNC, 5′-CCTAAGGTTAAGTCGCCCTCG-3′. Lentiviral *CXCR2* (NM_001557) overexpression particles were generated based on the pLenti-puro backbone. The recombinant lentiviral particles were co-transfected with lentivirus packaging systems (pLP1, pLP2, and pLP/VSVG plasmids) into 293FT cells. 48 h later, the supernatants were collected, filtered, and centrifuged. Endothelial cells were infected with lentivirus at an MOI of five.

### 4.5. Tube Formation Assay

In total, 300 μL of the Matrigel (Cat. No. 356231; BD Biosciences, San Diego, CA, USA) solution was added to each well of a 24-well plate. PUMC-HUVEC-T1 cells (3 × 104 cells/well) were then seeded on the Matrigel in the 24-well plate with TGF-β1 (5 ng/mL) for 24 h, either alone or in combination with CXCL8 (50 ng/mL). For specific groups, SB225002 (30 nM) or TPCA-1 (1 μM) were applied 2 h before CXCL8 treatment by the researchers. Tubular structures were examined using an inverted microscope (Nikon) and the number of tube branches in three fields.

### 4.6. Quantitative Reverse Transcription PCR (RT-qPCR)

RT-qPCR was performed as previously described [10]. The following primers were applied during amplification: CXCR2 forward, 5′-TCCGTCACTGATGTCTACCTGC-3′ and reverse, 5′-TCCTTCAGGAGTGAGACCACCT-3′; *SLC7A11*, forward (F), 5′-TCCTGCTTTGGCTCCATGAACG-3′ and reverse (R), 5′-AGAGGAGTGTGCTTGCGGACAT-3′; *GPX4*, F, 5′-ACAAGAACGGCTGCGTGGTGAA-3′ and R, 5′-GCCACACACTTGTGGAGCTAGA-3′; *HMOX1*, F, 5′-CCAGGCAGAGAATGCTGAGTTC-3′ and R, 5′-AAGACTGGGCTCTCCTTGTTGC-3′; *NRF2*, F, 5′-CACATCCAGTCAGAAACCAGTGG-3′ and R, 5′-GGAATGTCTGCGCCAAAAGCTG-3′; *TFR2*, F, 5′-GCACCTCAAAGCCGTAGTGTAC-3′ and R, 5′-CCACCTGTTCATAGAGAGTCTGC-3′; *CD31*, F, 5′-AAGTGGAGTCCAGCCGCATATC-3′ and R, 5′-ATGGAGCAGGACAGGTTCAGTC-3′; *CDH5*, F, 5′-GAAGCCTCTGATTGGCACAGTG-3′ and R, 5′- TTTTGTGACTCGGAAGAACTGGC-3′; *CD34*, F, 5′-CCTCAGTGTCTACTGCTGGTCT-3′ and R, 5′-GGAATAGCTCTGGTGGCTTGCA-3′; *S100A4*, F, 5′-CAGAACTAAAGGAGCTGCTGACC-3′ and R, 5′-CTTGGAAGTCCACCTCGTTGTC-3′; *ACTA2*, F, 5′-CTATGCCTCTGGACGCACAACT-3′ and R, 5′-CAGATCCAGACGCATGATGGCA-3′; CDH2, F, 5′-CCTCCAGAGTTTACTGCCATGAC-3′ and R, 5′-GTAGGATCTCCGCCACTGATTC-3′; and *ACTB*, F, 5′-CACCATTGGCAATGAGCGGTTC-3′ and R, 5′-AGGTCTTTGCGGATGTCCACGT-3′. Relative gene expression was calculated using the 2-ΔΔCq method, using ACTB as the control gene.

### 4.7. Western Blotting Assays

Western blotting was performed as previously described [17]. The following antibodies and dilutions were applied: anti-CXCR2 (1:1000, 20634-1-AP, Proteintech, Wuhan, China), anti-Phospho-IκBα (Ser32) (p-IκBα) (1:1000, #2859, Cell Signaling Technology, Danvers, MA, USA), anti-IκBα (1:1000, #4814, Cell Signaling Technology), anti-NF-κB p65 (1:1000, #8242, Cell Signaling Technology), anti-phospho-NF-κB p65 (Ser536) (p-p65) (1:1000, #3033, Cell Signaling Technology), anti-CD31 (1:2000, 11265-1-AP, Proteintech), anti-VE-cadherin (VE-cad, 1:1000, 27956-1-AP, Proteintech), anti-CD34 (1:1000, 14486-1-AP, Proteintech), anti-S100A4 (1:5000, 16105-1-AP, Proteintech), anti-α-SMA (1:2000, 14395-1-AP, Proteintech), anti-N-cadherin (N-cad, 1:2000, 22018-1-AP, Proteintech), anti-SLC7A11 (1:1000, 26864-1-AP, Proteintech), anti-GPX4 (1:2000, 67763-1-Ig, Proteintech), anti-HO-1 (1:1000, 10701-1-AP, Proteintech), anti-NRF2 (1:1000, 80593-1-RR, Proteintech), anti-TFR2 (1:1000, ab80194, Abcam, Cambridge, UK), or anti-β-tubulin (1:5000, 10094-1-AP, Proteintech), or anti-β-actin (1:1000, 20536-1-AP, Proteintech). The protein bands were visualized using BeyoECL Star reagent (Beyotime, Shanghai, China) and the Tanon 4600SF Chemiluminescent Imaging System (Tanon, Shanghai, China). The endogenous protein expression control used was β-actin.

### 4.8. Measurement of GSG/GSSG, Reactive Oxygen Species (ROS), Lipid ROS (MDA), and Cell Viability

To detect the GSG/GSSG ratio, ROS, lipid ROS production, and cell viability, commercial kits were purchased and used following the manufacturers’ instructions, including GSH/GSSG Ratio Detection Assay (S0053, Beyotime), ROS/Superoxide Detection Assay Kit (Cell-based) (ab139476, Abcam), Lipid Peroxidation (MDA) Assay Kit (S0131S, Beyotime), and CCK-8 Kit (Beyotime).

### 4.9. Detection of Lipid Peroxidation

C11- BODIPY 581/591 (Maokang Biotechnology, Shanghai, China)) was used to monitor lipid peroxidation. PUMC-HUVEC-T1 cells were cultured in a six-well plate. After 24 h of treatment with erastin (10 μM) alone or in combination with CXCL8 (50 ng/mL), cells were washed and then treated with 10 µM of C11-BODIPY 581/591 for 30 min at 37 °C and washed with PBS. The mean fluorescence intensity (MFI) was measured using a flow cytometer (LSRFortessa; BD, Franklin Lakes, NJ, USA) by recording BODIPY emission on channels FL1-H at 530 nm and FL2-H at 585 nm. Data were collected from at least 10,000 cells and analyzed using NovoExpress (v1.5.4, Agilent, Santa Clara, CA, USA). The experiment was conducted three times independently.

### 4.10. Statistical Analysis

GraphPad Prism 9.5.1 software (GraphPad Software, San Diego, CA, USA) was utilized for statistical analysis. The results were expressed as the mean ± SD from at least three technical replicates of three independent experiments. To perform multiple comparisons, a one-way analysis of variance (ANOVA) with Tukey correction was employed. An unpaired Welch’s *t*-test was used for comparisons between the two groups. The significance level was established at *p* < 0.05.

## 5. Conclusions

CXCL8, mainly secreted by tumor-associated macrophages, can activate the NF-κB signaling pathway in endothelial cells via a CXCR2-dependent manner. The CXCL8-CXCR2/NF-κB axis can enhance EndMT and activate *SLC7A11* and *GPX4* expression, protecting endothelial cells from ferroptosis. This mechanism helps expand our understanding of the acquired resistance to ferroptosis in the ovarian tumor microenvironment.

## Data Availability

Data is contained within the article.
